# Lead in Cocoa and Chocolate: Rankin and Flegal Respond

**Published:** 2006-05

**Authors:** Charley W. Rankin, A. Russell Flegal

**Affiliations:** Environmental Toxicology, WIGS, University of California, Santa Cruz, Santa Cruz, California, E-mail: rankin@etox.ucsc.edu

We thank Manton for his comments on our study (Rankin et al. 2005), and we hope that readers will be encouraged to read the entire article to place his correspondence in perspective. In our article (Rankin et al. 2005), we documented the orders of magnitude of increases in lead concentrations of processed cocoa and chocolate products over that of the lead concentration in cocoa bean nibs used in the manufacture of those products. We maintain that our conclusions are substantiated by both the data and the extensive literature documenting industrial lead contamination in the biosphere (Cocoa Producers’ Alliance 2004; National Research Council 1993]. Our lead concentration measurements show that manufactured cocoa and chocolate products exhibit contamination not found in the source material—cocoa bean nibs. As detailed in the article, the average lead concentration of the cocoa bean nibs was among the lowest reported values for a natural food, whereas the lead concentrations of the cocoa products were up to over 300-fold higher; as we noted in our article (Rankin et al. 2005), those relatively high levels of lead have been acknowledged by the Cocoa Producers’ Alliance (Lagos, Nigeria).

Because the concentration measurements indicated that this contamination was not naturally derived, we analyzed other materials for lead concentrations and lead isotopic composition to determine possible sources of the increase in lead in cocoa products. We are unsure about the origination of additional points in Figures 2 and 4, but we used the data listed in Table 3 in forming our original conclusions, and we continue to support those conclusions. To suggest, as Manton does, that the lead isotopic composition measurements show that the contamination is naturally derived is disingenuous and inconsistent with the extensive literature using lead isotopic compositions to characterize potential sources—natural and industrial—of lead in the biosphere (Cocoa Producers’ Alliance 2004; National Research Council 1993). We maintain that the lead isotopic composition measurements suggest that, although it may be possible for some contamination originating at the cocoa farms to be transferred to the final products, the majority of contamination must accumulate during shipping and/or manufacturing, as both the lead concentrations and lead isotopic compositions of cocoa and chocolate products indicate.

Finally, we are disappointed with Manton’s comment regarding our declaration of no competing financial interests. In our article (Rankin et al. 2005), we listed the American Environmental Safety Institute (AESI) as a source of funding. The institute provided some of the funding for the chemical analyses, scientific interpretation, and litigation depositions, but these were all done with the agreement that our study would be equipoised—conducted without bias. Moreover, the litigation was settled well before our manuscript was written, and we neither received nor requested funding from the AESI or any other organization or any individual involved in the litigation for the preparation or publication of the article.
